# Role of guanylate-binding protein 1 in the proliferation of invasive lung adenocarcinoma cells

**DOI:** 10.3389/fonc.2025.1434249

**Published:** 2025-02-13

**Authors:** Takashi Kumada, Takahiro Mimae, Norifumi Tsubokawa, Kei Kushitani, Yukio Takeshima, Yoshihiro Miyata, Morihito Okada

**Affiliations:** ^1^ Department of Surgical Oncology, Hiroshima University, Hiroshima, Japan; ^2^ Department of Pathology, Hiroshima University, Hiroshima, Japan

**Keywords:** GBP1, lung adenocarcinoma, prognostic factor, inhibitor, cell growth, therapy

## Abstract

**Background:**

Guanylate-binding protein 1 (GBP1) is involved in the malignant progression of lung adenocarcinoma, particularly in the acquisition of invasive potential. However, its role in tumor proliferation and therapeutic viability in invasive lung adenocarcinomas remains unclear.

**Methods:**

This study included 99 patients with invasive lung adenocarcinoma, excluding those with non-invasive lepidic components, who had undergone complete pulmonary resection. Immunohistochemical staining was performed to examine the presence of GBP1, and its prognostic significance was assessed using uni- and multi-variable Cox regression analyses. Additionally, the expression levels of GBP1 gene and protein levels were evaluated in lung adenocarcinoma cell lines (PC-9, A549, NCI-H322, NCI-H441, NCI-H820, and ABC-1), and its proliferative role in these cell lines was analyzed using specific inhibitors targeting GBP1.

**Results:**

GBP1 expression was detected in 45 (45.5%) patients. The 5-year overall survival rates for GBP1-positive and -negative patients were 66.0% (95% confidence interval (CI): 46.3–80.0%) and 85.7% (95% CI: 72.0–93.0%), respectively (P = 0.029). The multivariable analysis demonstrated that GBP1 positivity was an independent factor for poor overall survival (hazard ratio [HR] = 2.52 [95% CI: 1.02–6.22], P = 0.045). GBP1 gene and protein were markedly expressed in NCI-H820 than in NCI-H322 and ABC-1. The inhibitor targeting GBP1 significantly suppressed the growth of NCI-H820 but not that of NCI-H322 or ABC-1.

**Conclusions:**

GBP1 is a prognostic factor that may be involved in the proliferation of invasive lung adenocarcinoma, suggesting that inhibiting GBP1 activity may be a promising therapeutic approach for lung adenocarcinoma patients expressing GBP1.

## 1 Introduction

Lung adenocarcinoma is the major histological type of non-small cell lung cancer characterized by the presence of both non-invasive and invasive components (1), making it an ideal biological model for studying tumor progression within the same lesion during the same period. Invasive adenocarcinomas exhibit diverse oncogenic mechanisms and tumor properties, with a higher malignant potential than those with non-invasive components (2, 3).

In our previous study, cancer cells were selectively collected from the non-invasive and invasive components of lung adenocarcinoma using laser microdissection. and the non-invasive and invasive components were compared (4). Guanylate-binding protein 1 (GBP1) was identified as a candidate molecule that was highly expressed in the invasive component. Furthermore, GBP1 promotes the migratory ability of non-invasive lung adenocarcinoma cells, indicating its potential role in invasion (4, 5). In addition, GBP1 expression was detected in resected specimens from patients with lung adenocarcinoma using immunohistochemistry, and GBP1 positivity was correlated with lymphatic vessel invasion (5).

Although the role of GBP1 in the malignant progression of non-invasive lung adenocarcinoma cells have been documented (5), the expression status of GBP1 and its role in invasive adenocarcinoma remain unclear. Additionally, the possibility of targeting GBP1 for treating GBP1-positive lung adenocarcinoma cells has not been elucidated.

Therefore, immunohistochemical examinations of human surgical specimens of pure invasive lung adenocarcinoma were performed in this study to evaluate GBP1 expression in invasive lung adenocarcinoma cells and investigate its association with clinicopathological features. Furthermore, using inhibitors targeting GBP1 in human lung adenocarcinoma cell lines, the role of GBP1 on invasive adenocarcinoma cell proliferation was evaluated.

## 2 Materials and methods

### 2.1 Study cohort

Between April 2013 and March 2017, 99 patients with lung adenocarcinoma without lepidic, that is, non-invasive components, who had undergone pulmonary resections at Hiroshima University Hospital were included in this study. Those patients who received induction therapy with the non-availability of adequate hematoxylin-eosin stained pathological slides were excluded. The Institutional Review Boards of the participating institutions approved this retrospective review of a prospective database, and the requirement for informed consent was waived (approval no. March 28, 2016, E294; August 22, 2023, E 2023-0104).

### 2.2 GBP1 immunostaining

**Figure 1:**
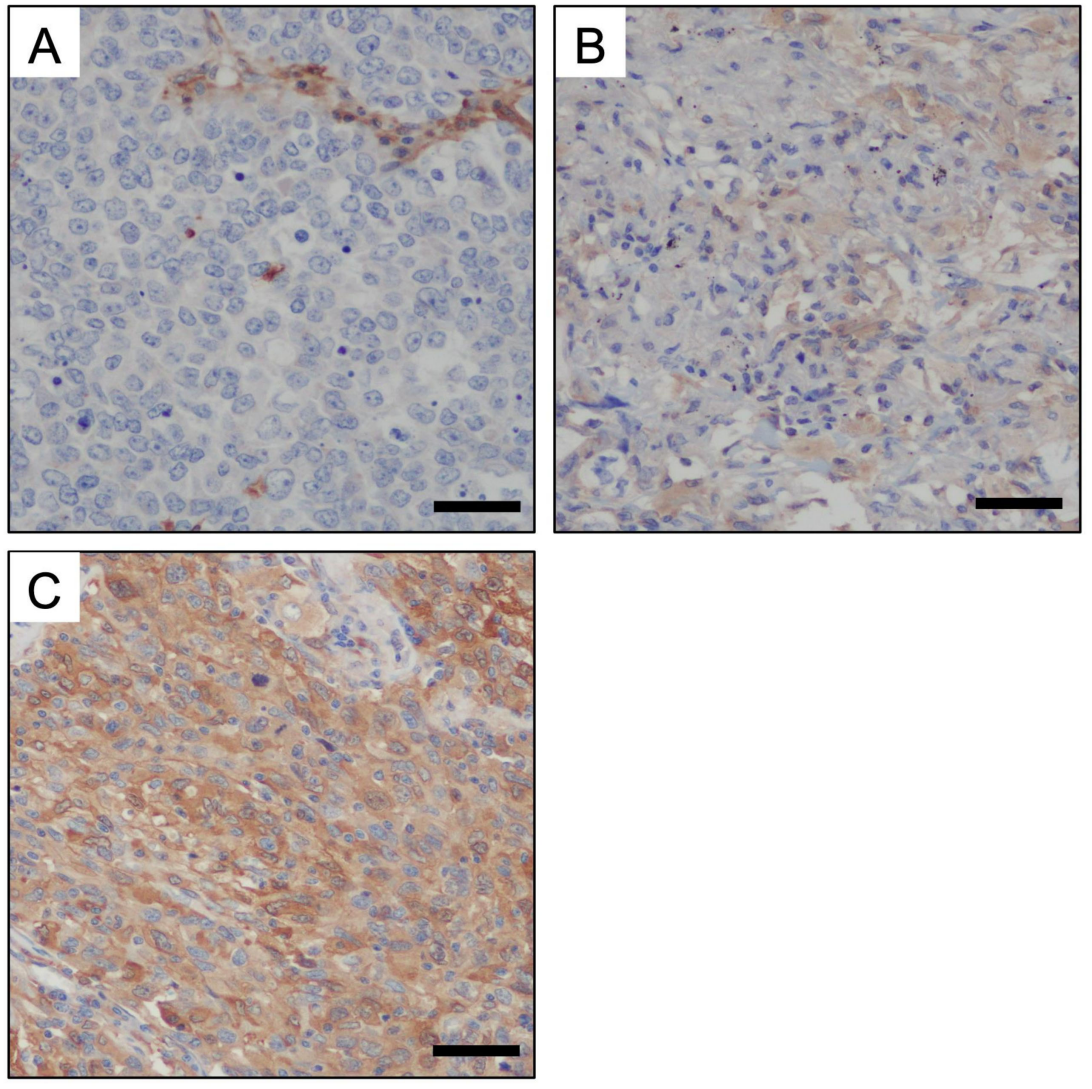
Immunohistochemical staining for GBP1 on human invasive adenocarcinoma tissue. The staining intensity for GBP-1 was divided into the following three categories: non staining **(A)**, weak staining **(B)**, and strong staining **(C)**. Strong staining indicated GBP1 positivity (Scale bars: 50 μm).

Formalin-fixed and paraffin-embedded blocks were sectioned at 4 mm thickness. For immunohistochemical staining targeting GBP1, Ventana Benchmark GX (Roche, USA) was used with an ultraView Universal DAB Detection Kit (#760-500; Ventana, USA) and anti-GBP1 (1:1500; Abcam, ab131255, U.K) antibodies. The staining intensity was divided into three categories: none, weak, and strong. The presence of a strong signal in any area of the tumor cells indicated positive immunohistochemical staining (**Figure 1**).

### 2.3 Statistical analysis

Continuous and categorical variables were analyzed using nonparametric Mann–Whitney U tests, Kruskal–Wallis tests and χ2 or Fisher exact tests. Overall survival (OS) was defined as the interval between surgery and death due to any cause. Recurrence-free survival (RFS) was defined as the interval between surgery and the first event (relapse or death from any cause). Postoperatively, most patients underwent chest plain HRCT scans and blood tests every six months. OS and RFS rates were calculated from the Kaplan–Meier curves, and two or more groups were compared using univariable log-rank analysis. Prognostic factors were determined by multivariable Cox regression analysis of OS and RFS. The following variables were included in the multivariable Cox model: age; sex; presence or absence of lymphatic, venous, or pleural invasion; smoking status; lobectomy; and staging. All statistical analyses were performed using EZR (Saitama Medical Center, Jichi Medical University, Saitama, Japan), which is a graphical user interface for R (The R Foundation for Statistical Computing, Vienna, Austria) (6). More precisely, it is a modified version of the R Commander, designed to add statistical functions frequently used in biostatistics.

### 2.4 Cell culture

The lung cancer cell lines A549, NCI-H322, NCI-H441, and NCI-H820 were purchased from ATCC, PC-9 cells from Riken Cell Bank, and ABC-1 cells from JCRB. PC-9, NCI-H322, NCI-H441, and NCI-H820 cells were maintained in Roswell Park Memorial Institute 1640 medium, A549 cells in Dulbecco’s modified Eagle’s medium, and ABC-1 cells in Eagle’s minimum essential medium. The media were supplemented with 10% fetal bovine serum. All cells were cultured at 37°C in a humidified atmosphere of 5% CO2.

### 2.5 Reverse transcription-polymerase chain reaction analysis

Total RNA was extracted from the lung adenocarcinoma cell lines PC-9, A549, NCI-H322, NCI-H441, NCI-H820, and ABC-1 using the TRI Reagent (Molecular Research Center, Inc.), and cDNA was synthesized using a High-Capacity cDNA Reverse Transcription Kit (Applied Biosystems; Thermo Fisher Scientific, Inc.) according to the manufacturer’s protocol. Quantitative PCR was performed using TaqMan Universal PCR Master Mix (Applied Biosystems; Thermo Fisher Scientific, Inc.) and TaqMan Gene Expression Assays (Hs00977005_m1 GBP1; Thermo Fisher Scientific, Inc.). Finally, PCR was performed under the following conditions: 10 min at 95°C, followed by 40 cycles of 15 s at 95°C and 1 min at 60°C. The results were analyzed with the ΔΔCq method.

### 2.6 Western blot analysis

Whole-cell extracts were prepared using RIPA buffer supplemented with phenylmethylsulfonyl fluoride and protease inhibitors. Protein lysates were resolved using sodium dodecyl sulfate-polyacrylamide gel electrophoresis, transferred to polyvinylidene fluoride membranes, and incubated with primary antibodies. Antibodies against β-actin (1:15,000; Merck, A5441, USA) and GBP1 (1:1,500; Abcam, ab131255, U.K) were used in this study. After blocking with PVDF Blocking Reagent for Can Get Signal (Toyobo, NYPBR01, Japan) for 2 h at room temperature, the immunoreactive proteins were visualized by the ECL Select™ Western Blotting Detection System (Cytiva, Tokyo, Japan).

### 2.7 Cell proliferation analysis

The cell proliferation assay was performed using a CyQUANT Cell Proliferation Kit (Molecular Probes, Eugene, OR, USA). Cells were seeded on 96-well plates with 200 µL of the complete medium. A total of 500 cells of PC-9, A549, and NCI-H820, 1,000 cells of NCI-H322 and NCI-H441, and 2,000 of ABC-1 per well were used in this assay. After 24 h of incubation, the medium was changed to a complete medium with 1 µM of GBP1 inhibitor (NSC756093, Sigma, SML1310, USA) dissolved in DMSO. The control group was changed to a medium with the same amount of DMSO to the inhibitor. The cells were then incubated for 144 h, following which the medium was aspirated and the plates were frozen at -80°C. The cells were then lysed with the lysis buffer from the CyQUANT kit. The fluorescence intensity of the DNA-binding dye was measured using a microplate reader at excitation and emission wavelengths of 485 and 520 nm, respectively.

## 3 Results

### 3.1 Baseline and postoperative characteristics in patients with lung adenocarcinoma according to GBP1 expression status

**Table 1** summarizes the characteristics of the included patients. This study included 99 patients with invasive lung adenocarcinoma (63 [63.6%] men and 36 [36.4%] women; median age 69 years, interquartile range [IQR]: 64–73) The number of patients who smoked more than 40 pack-year was 45 (45.5%). The median whole-tumor size on high-resolution computed tomography (HRCT) was 2.0 (1.5–3.2) cm. The median solid tumor size on HRCT was 1.8 (1.5–3.2) cm. The numbers of patients with clinical stages I, II, and III were 78 (77.2%), 17 (16.8%), and 4 (4.0%), respectively. The median pathological tumor size was 2.1 (1.5–3.5) cm (**Table 2**). Pathological stages I, II, III, and IV were found in 69 (69.7%), 15 (15.2%), 14 (14.1%), and 1 (1.0%) patients, respectively. No significant difference in baseline characteristics was observed between GBP1-positive and negative patients.

**Table 1:** Baseline characteristics.

Variables	Total	GBP1 expression		*P*-Value
	n=99, n (%)	(-) n=54, n (%)	(+) n=45, n (%)	
Age (years) Median(IQR)	69 (64–73)	68 (64–72)	70 (65–74)	0.56
Sex				1.00
Male	63 (63.6)	34 (63.0)	29 (64.4)	
Female	36 (36.4)	20(37.0)	16 (35.6)	
Smoking status (> 40 pack-years)	45 (45.5)	20 (37.0)	25 (55.6)	0.07
CT whole tumor size (cm) Median (IQR)	2.0 (1.5–3.2)	1.8 (1.4–3.3)	2.1 (1.6–3.1)	0.36
CT solid tumor size (cm) Median (IQR)	1.8(1.5–3.2)	1.7(1.3–3.3)	2.1(1.6–3.1)	0.15
SUVmax Median (IQR)	3.5 (1.9–7.1)	3.2 (1.6–6.8)	3.8 (2.3–7.9)	0.17
cT				0.68
cT 1	64 (64.6)	36 (66.7)	28 (62.2)	
cT 2 - 4	35 (35.4)	18 (33.3)	17 (37.8)	
cN				0.13
cN 0	87 (87.9)	50 (92.6)	37 (82.2)	
cN 1 or 2	12 (12.1)	4 (7.4)	8 (17.8)	
cStage				0.32
cStage I	78 (78.8)	45 (83.3)	33 (73.3)	
cStage II or III	21 (21.2)	9 (16.7)	12 (26.7)	

GBP1, guanylate-binding protein 1; CT, computed tomography; SUV, standardized uptake value; IQR, interquartile range.

**Table 2:** Postoperative characteristics of the participants.

Variable	Total	GBP1-Expression		*P*-Value
	n=99, n (%)	(-) n=54, n (%)	(+) n=45, n (%)	
Pathological size (cm) Median (IQR)	2.1 (1.5–3.5)	1.9 (1.5–3.4)	2.5 (1.5–3.5)	0.27
Ly(+)	30 (30.3)	17 (31.5)	13 (28.9)	0.83
V(+)	42 (42.4)	20 (37.0)	22 (48.9)	0.31
PL(+)	30 (30.3)	14 (25.9)	16 (35.6)	0.38
PM(+)	4 (2.0)	2 (3.7)	2 (4.4)	1.00
Predominant subtype				0.22
Acinar	9 (8.9)	4 (7.4)	5 (11.1)	
Papillary	63 (63.6)	36 (66.7)	27 (60.0)	
Micropapillary	6 (6.1)	5 (9.3)	1 (2.2)	
Solid	19 (19.2)	7 (13.0)	12 (26.7)	
Invasive mucinous adenocarcinoma	1 (1.0)	1 (1.9)	0 (0)	
Colloid	1 (1.0)	1 (1.9)	0 (0)	
pT				0.42
pT 1	51 (50.5)	30 (55.6)	21 (46.7)	
pT 2–4	48 (49.5)	24 (44.4)	24 (53.3)	
pN				0.32
pN 0	78 (78.8)	45 (83.3)	33 (73.3)	
pN 1 or 2	21 (21.2)	9 (16.7)	12 (26.7)	
pM				1.00
pM 0	98 (99.0)	53 (98.1)	45 (100)	
pM 1	1 (1.0)	1 (1.9)	0 (0)	
pStage				0.38
pStage I	69 (69.7)	40 (74.1)	29 (64.4)	
pStage II - IV	30 (30.3)	14 (25.9)	16 (35.6)	

GBP1, guanylate-binding protein 1; Ly, lymphatic invasion; V, venous invasion; PL, pleural invasion; PM, pulmonary metastasis; IQR, interquartile range; CI, confidence interval.

Of the 30 patients (30.3%) with lymphatic invasion, 13 (28.9%) and 17 (31.5%) were GBP1-positive and negative, respectively (P = 0.83). Vascular invasion was present in 42 patients (42.4%), among whom 22 (48.9%) and 20 (37.0%) were GBP1-positive and negative, respectively (P = 0.31).

Regarding the predominant subtypes of lung adenocarcinoma, 63 patients had papillary adenocarcinoma (27 [60.0%] were GBP1-positive), 19 had solid type (12 [26.7%] were GBP1-positive), 9 had acinar type (5 [11.1%] were GBP1-positive), and 6 had micropapillary type (1 patient [2.2%] was GBP1-positive) (P = 0.22).

### 3.2 Prognosis in patients with lung adenocarcinoma after surgical resections according to the GBP1 expression status

The prognosis, including OS and RFS, of patients with lung adenocarcinoma who underwent pulmonary resection was evaluated.

**Figure 2:**
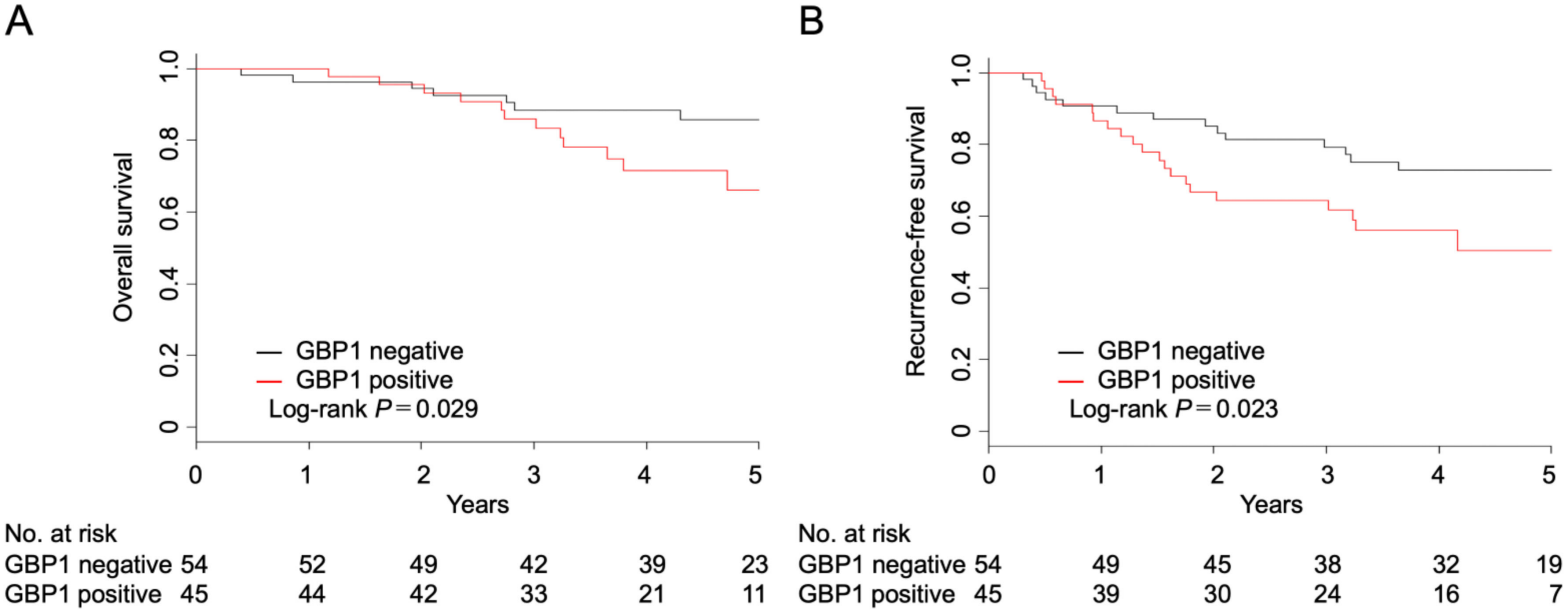
Overall **(A)** and recurrence-free survival **(B)** after complete resection in patients with invasive adenocarcinoma according to the GBP1 status.

The 5-year OS rates for GBP1-positive and -negative patients were 66.0% (95% confidence interval (CI): 46.3–80.0%) and 85.7% (95% CI: 72.0–93.0%), respectively (P = 0.029) (**Figure 2A**). The 5-year RFS rates for GBP1-positive and negative patients were 50.5% (95% CI: 32.6–65.9%) and 72.8% (95% CI: 58.3–83.0%), respectively (P = 0.023) (**Figure 2B**).

### 3.3 Factors affecting RFS and OS in patients with lung adenocarcinoma

To further detect the correlation between GBP1 expression and OS and RFS in patients with lung adenocarcinoma who had undergone surgical resection, multivariable Cox proportional hazards regression analysis was performed (**Table 3**; **Supplementary Table 1**). GBP1 positivity was an independent factor for reduced OS (HR = 2.52 [95% CI: 1.02–6.22], P = 0.045) and RFS (HR = 1.98 [95% CI: 0.96–4.05], P = 0.063). In addition, increased age (> 70 years) was associated with reduced OS (HR = 4.15 [95% CI: 1.49–11.56], P = 0.0064) and RFS (HR = 2.87 [95% CI: 1.33–6.17], P = 0.0071).

**Table 3:** Univariate and multivariable cox regression analysis for overall survival.

	Overall survival			
	Univariate analysis		Multivariable analysis	
	Hazard ratio (95% CI)	P-value	Hazard ratio (95% CI)	P-value
Sex (Male)	1.43 (0.58–3.51)	0.43	2.36 (0.79–7.04)	0.12
Age (> 70 years)	2.94 (1.15–7.53)	0.024	4.15 (1.49–11.56)	0.0064
Smoking status (> 40 pack-years)	0.90 (0.38–2.10)	0.80	0.49 (0.18–1.31)	0.15
Surgical method (Lobectomy)	1.54 (0.60–3.96)	0.37	1.19 (0.37–3.80)	0.77
Ly, V, or PL (+)	1.68 (0.68–4.13)	0.26	1.29 (0.49–3.40)	0.60
p Stage (II–IV)	2.34 (1.01–5.43)	0.048	2.53 (0.90–7.12)	0.077
GBP1-positive	2.56 (1.07–6.15)	0.035	2.52 (1.02–6.22)	0.045

GBP1, guanylate-binding protein 1; Ly, lymphatic invasion; V, venous invasion; PL, pleural invasion; CI, confidence interval.

### 3.4 Gene and protein expression status of GBP1 in human lung adenocarcinoma cell lines and growth suppression by GBP1 inhibitor

**Figure 3:**
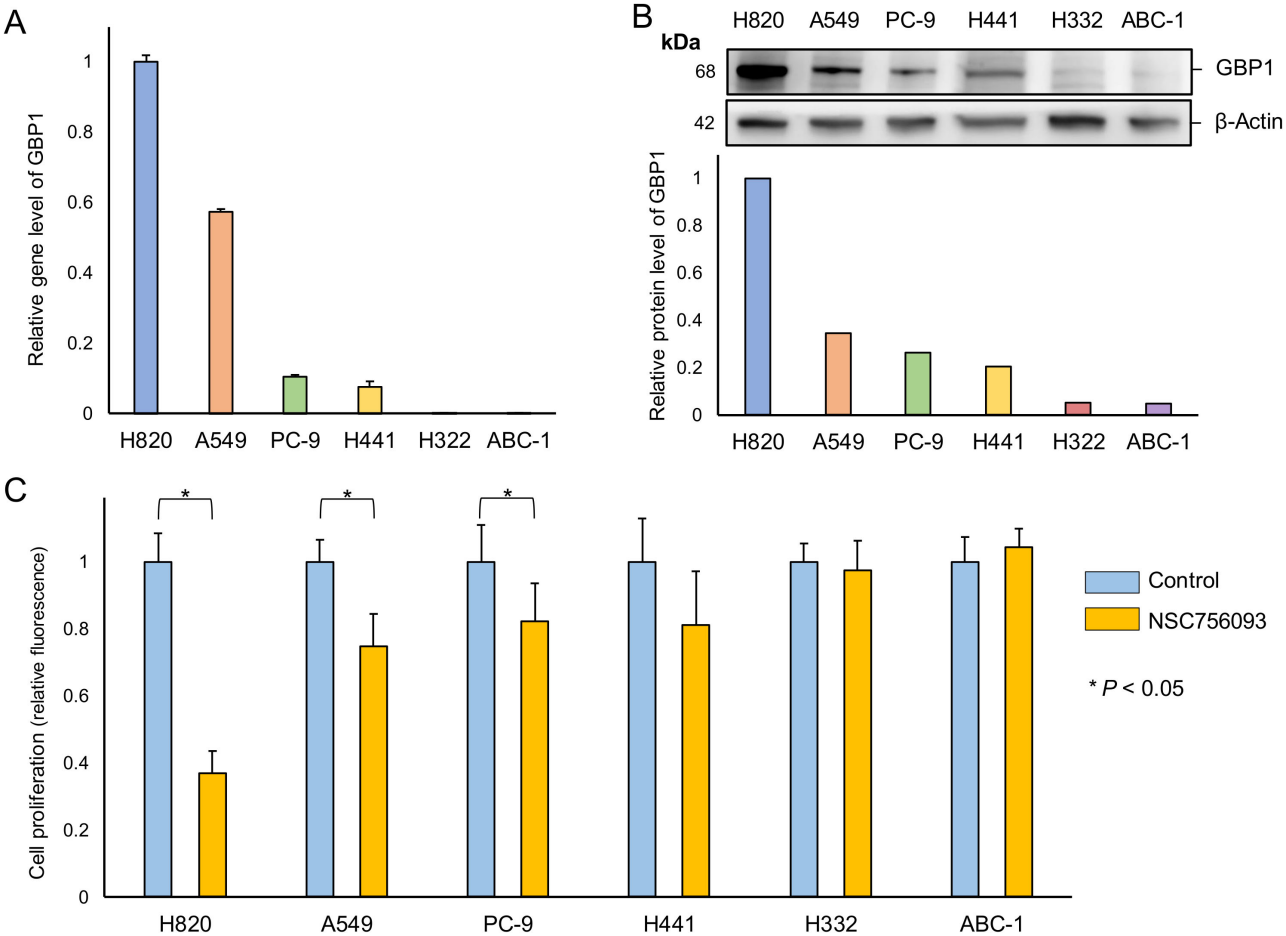
Relationship between GBP1 gene and protein expression and proliferative potential in lung adenocarcinoma cells. **(A)** Total RNAs were extracted from each cell and analyzed by RT-PCR using primer sets for GBP1 and ß-actin. Using the ΔΔCt method, the relative expression levels of GBP-1 to ß-actin in each lung adenocarcinoma cell line are shown with H820 as the calibrator. **(B)** Total protein was extracted from each cell and analyzed by western blotting using antibodies for GBP1 and ß-actin. The relative expression of GBP1 to ß-actin was determined in each cell line with H820 as a calibrator. **(C)** In each cell line, the control and the inhibitor (NSC756093) groups were cultured for 6 days. The control group served as the calibrator, and the relative cell quantity of the inhibitor group was calculated.

RT-PCR demonstrated that the strongest expression of GBP1 in lung adenocarcinoma cell lines was observed in NCI-H820, followed by A549, whereas the gene expression for GBP1 was lower in PC-9 and NCI-H441. In contrast, NCI-H322 and ABC-1 showed only modest GBP1 expression (**Figure 3A**).

Consistent with these results, western blotting of the GBP1 protein in lung adenocarcinoma cell lines revealed similar findings, with the strongest expression in NCI-H820 and weak expression in A549 cell lines. PC-9 and NCI-H441 cell lines demonstrated minimal GBP1 protein expression, and NCI-H322 and ABC-1 cell lines demonstrated modest expression of GBP1 (**Figure 3B**).

The addition of GBP1 inhibitor to the culture medium of NCI-H820 cells substantially inhibited cell growth. In addition, the growth of A549 cells was suppressed, whereas the effect of the inhibitor on cell proliferation was less than in NCI-H820 cell lines. GBP1 inhibitor-induced growth suppression was less in PC-9 and NCI-H441 than in A549 cell lines. Moreover, GBP1 inhibitor exerted a negligible effect on growth suppression in NCI-H322 and ABC-1 cell lines (**Figure 3C**).

## 4 Discussion

To our knowledge, this is the first study to highlight the role of GBP1 in promoting the proliferation of human lung adenocarcinoma cells. Approximately 50% of the lung adenocarcinomas without non-invasive components were positive for GBP1, in contrast to previous findings that demonstrated that only 12.5% of the tumors were positive in patients with lung adenocarcinomas containing non-invasive components (5). However, the frequency of lymphatic or venous invasion was comparable between patients with GBP1-positive and -negative tumors, which also differed from the results of a previous study (5). In addition, multivariable analyses of both OS and RFS revealed that GBP1 was an independent prognostic factor. GBP1 contributes to the acquisition of invasive potential in non-invasive lung adenocarcinoma (5); however, our findings elucidate that GBP1 is involved in promoting another type of malignant progression in lung adenocarcinoma cells after acquiring invasive potential, which may be attributed to the proliferative ability. This finding suggests that GBP1 plays a significant role in tumor progression and patient outcomes. However, we acknowledge that the observed statistical associations may have been influenced by additional confounding factors that were not fully accounted for in our analysis. Factors such as histological subtype, postoperative adjuvant therapy, and other molecular alterations may also contribute to survival outcomes. Addressing these factors requires larger multicenter studies with diverse patient cohorts to validate our findings and ensure a more comprehensive understanding of the biological and clinical significance of GBP1.

GBP1 is a large GTP-binding protein with GTPase activity (7). GTPases bind to GTP and hydrolyze it to GDP. GTPases play important roles in cell proliferation, cell differentiation, signal transduction, and intracellular protein transport (8, 9). GBP1 regulates migration and invasion of oral squamous cell carcinoma *in vitro* (10). GBP1 expression is a significant prognostic factor of PFS in patients with ovarian cancer (11, 12). Furthermore, GBP1 expression is positively correlated with metastasis and progression to brain metastasis in patients with breast cancer (13). In addition, GBP1 is involved in cell proliferation in triple-negative breast cancer (14), consistent with our results. Based on these findings and the prognostic outcomes based on the GBP1 expression profile in our study, GBP1 may also be a potential therapeutic target for other types of cancer.

GBP1 gene and protein expressions were confirmed in several human lung adenocarcinoma cell lines, and differences in relative expression levels were observed between these cell lines. This expression pattern showed parallel results between the gene and protein levels. Additionally, a GBP1 inhibitor assay revealed a positive correlation between GBP1 expression and the suppressive effect of the inhibitor on cell growth. These findings imply that GBP1 is expressed in some human lung adenocarcinoma cells, and GBP1 inhibitors could potentially suppress cell proliferation in patients with GBP1-positive lung adenocarcinomas. The GBP1 inhibitor (NSC756093) used in this study binds to GBP1 and inhibits interaction with PIM1 (15). PIM1 regulates MYC transcriptional activity, cell cycle regulation, phosphorylation, and apoptotic protein inhibition, contributing to carcinogenicity (16). PIM1 promotes tumor growth in prostate cancer by interacting with MYC (17). Both PIM1 knockdown and PIM1 inhibitors decrease proliferation in triple-negative breast cancer (18). GBP1 may enhance signaling and proliferation by interacting with PIM1. Therefore, the binding of NSC756093 to GBP1 may alter the GBP1-PIM1 interaction by altering the 3D structure of GBP1, leading to growth inhibition.

GBP1 is regulated by the p53 and YY1 transcription factors, with p53 binding upstream promoter sites and YY1 is activated by p38-MAPK. Both pathways respond to cellular stressors, such as DNA damage, TNFα, and TGFβ, coordinating cell cycle arrest, reduced apoptosis sensitivity, and stress response activation. GBP1 likely promotes resistance to apoptosis under various stresses while enabling cell survival under harsh conditions (19). In cervical cancer, GBP1, after binding to HNRNPK, regulates CD44 protein expression through alternative splicing at the 3′ splice site, ultimately playing a cancer-promoting role (20). These findings suggest that GBP1 utilizes similar mechanisms in lung adenocarcinoma, interacting with splicing factors or stress response pathways to regulate tumor progression. However, further studies are required to confirm this hypothesis.

Adenocarcinomas with and without non-invasive cancer components have distinct clinical phenotypes and behaviors (2, 3, 21). Although GPB1 promotes malignant progression in both types of adenocarcinoma, the specific role of GBP1 differs depending on the stage of malignant progression of lung adenocarcinoma. Conversely, GBP1 may contribute to the acquisition of invasion ability in non-invasive cancer cells (5), whereas it contributes to the promotion of cell proliferation in invasive cancer cells, that is, cells after acquiring invasiveness.

Our study had certain limitations. First, the lack of *in vivo* experiments or research results introduces uncertainty regarding the validity of GBP1 as a potential target for treating human lung adenocarcinoma. Furthermore, the predictive analysis was based on retrospective data, and the results were not definitive. Nevertheless, based on the results of the *in vitro* experiments in this study, GBP1 plays a crucial role in the malignant progression of lung adenocarcinoma.

In conclusion, GBP1 may be involved in the proliferation of invasive lung adenocarcinomas. Therefore, inhibiting the activity of GBP1 may be a promising therapeutic approach for patients with lung adenocarcinomas expressing GBP1. Further studies are necessary to validate the results of this study and expand the indications of GBP1 inhibitors as human therapeutics for lung adenocarcinoma.

## Data Availability

The original contributions presented in the study are included in the article/**Supplementary Material**. Further inquiries can be directed to the corresponding authors.
