# Supplementary material for: Role of guanylate-binding protein 1 in the proliferation of invasive lung adenocarcinoma cells
Source: Front Oncol. 2025 Feb 13;15:1434249. doi: 10.3389/fonc.2025.1434249 (PMC11865198; doi:10.3389/fonc.2025.1434249)
Supplement: Supplementary file 1 [file DataSheet1.docx]

Supplementary Material

# Supplementary Figure legend

**Supplemental Figure 1.** Flow diagram of participant selection (patients with nonlepidic lung adenocarcinoma who had undergone surgery)

# Supplementary Table

| **Supplemental Table 1. Univariate and multivariate Cox regression analyses for recurrence-free survival** | | | | | |
| --- | --- | --- | --- | --- | --- |
|  | **Recurrence-free survival** | | | | |
|  | **Univariate analysis** | |  | **Multivariate analysis** | |
|  | **Hazard ratio (95% CI)** | **P-value** |  | **Hazard ratio (95% CI)** | **P-value** |
| **Sex (Male)** | 1.39 (0.68–2.84) | 0.36 |  | 2.03 (0.83–4.98) | 0.12 |
| **Age (˃ 70 years)** | 2.15 (1.07–4.33) | 0.031 |  | 2.87 (1.33–6.17) | 0.0071 |
| **Smoking status (˃ 40 pack-years)** | 1.11 (0.57–2.16) | 0.76 |  | 0.57 (0.26–1.29) | 0.18 |
| **Surgical method (Lobectomy)** | 2.99 (1.31–6.87) | 0.0097 |  | 2.16 (0.82–5.73) | 0.12 |
| **Ly, V, or PL (+)** | 2.37 (1.11–5.07) | 0.026 |  | 1.70 (0.74–3.91) | 0.21 |
| **p Stage (II–IV)** | 3.67 (1.88–7.16) | <0.001 |  | 2.65 (1.20–5.84) | 0.016 |
| **GBP1-positive** | 2.17 (1.10–4.28) | 0.026 |  | 1.98 (0.96–4.05) | 0.063 |
| GBP1, guanylate-binding protein 1; Ly, lymphatic invasion; V, venous invasion; PL, pleural invasion; CI, confidence interval. | | | | | |
