# Supplementary figures and images for: Role of guanylate-binding protein 1 in the proliferation of invasive lung adenocarcinoma cells
Source: Front Oncol. 2025 Feb 13;15:1434249. doi: 10.3389/fonc.2025.1434249 (PMC11865198; doi:10.3389/fonc.2025.1434249)

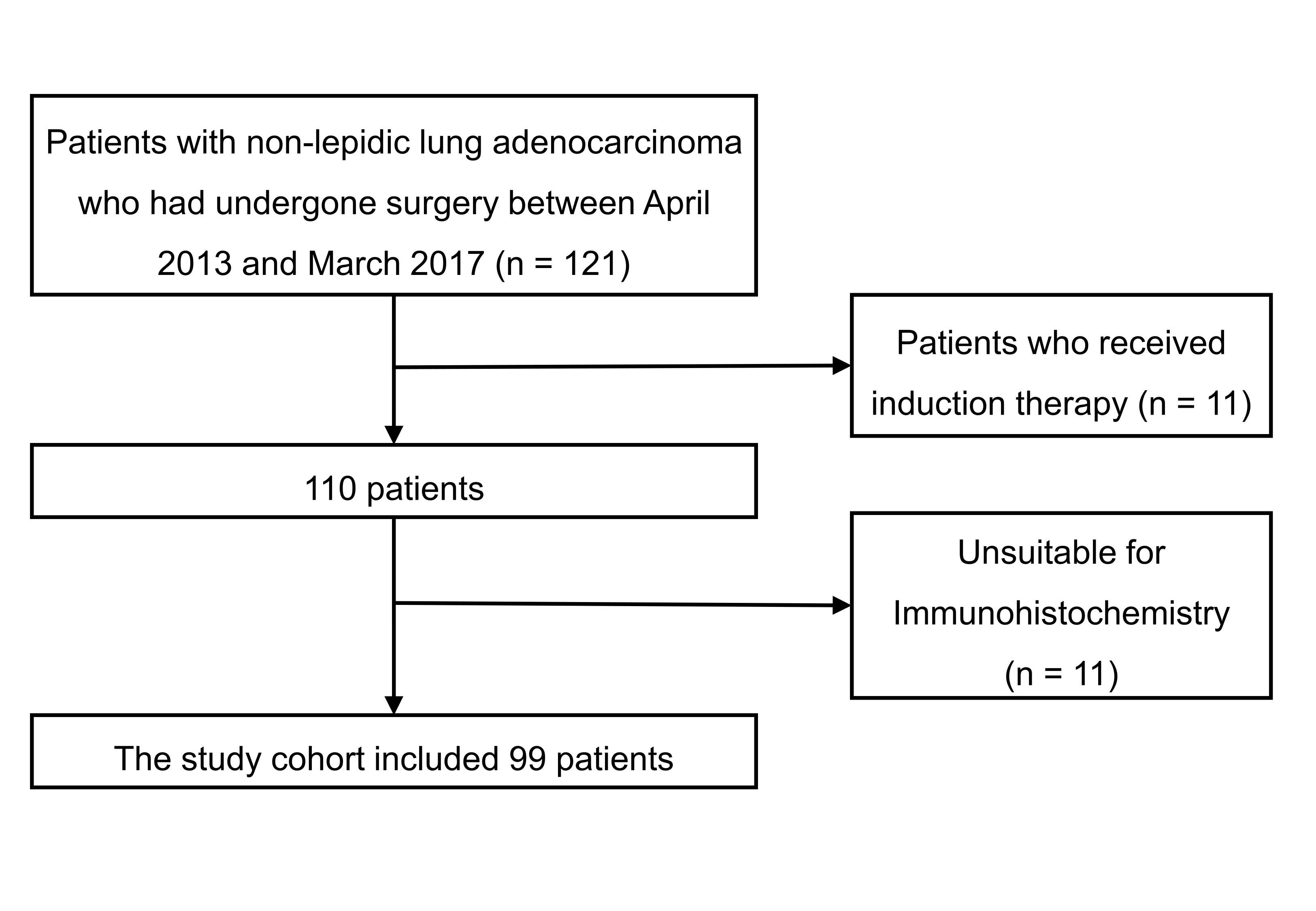

Supplement: Supplementary file 2 [file Image1.tif]
